# Proliferation and survival molecules implicated in the inhibition of BRAF pathway in thyroid cancer cells harbouring different genetic mutations

**DOI:** 10.1186/1471-2407-9-387

**Published:** 2009-10-31

**Authors:** Ana Preto, Joana Gonçalves, Ana P Rebocho, Joana Figueiredo, Ana M Meireles, Ana S Rocha, Helena M Vasconcelos, Hugo Seca, Raquel Seruca, Paula Soares, Manuel Sobrinho-Simões

**Affiliations:** 1 Institute of Molecular Pathology and Immunology of the University of Porto (IPATIMUP), 4200-465, Porto, Portugal; 2 Department of Biology, University of Minho, Campus de Gualtar, 4710-057 Braga, Portugal; 3 Faculty of Pharmacy of the University of Porto, 4050-047 Porto, Portugal; 4 Medical Faculty of the University of Porto, 4200-465 Porto, Portugal

## Abstract

**Background:**

Thyroid carcinomas show a high prevalence of mutations in the oncogene BRAF which are inversely associated with RAS or RET/PTC oncogenic activation. The possibility of using inhibitors on the BRAF pathway as became an interesting therapeutic approach. In thyroid cancer cells the target molecules, implicated on the cellular effects, mediated by inhibition of BRAF are not well established. In order to fill this lack of knowledge we studied the proliferation and survival pathways and associated molecules induced by BRAF inhibition in thyroid carcinoma cell lines harbouring distinct genetic backgrounds.

**Methods:**

Suppression of BRAF pathway in thyroid cancer cell lines (8505C, TPC1 and C643) was achieved using RNA interference (RNAi) for BRAF and the kinase inhibitor, sorafenib. Proliferation analysis was performed by BrdU incorporation and apoptosis was accessed by TUNEL assay. Levels of protein expression were analysed by western-blot.

**Results:**

Both BRAF RNAi and sorafenib inhibited proliferation in all the cell lines independently of the genetic background, mostly in cells with BRAF^V600E ^mutation. In BRAF^V600E ^mutated cells inhibition of BRAF pathway lead to a decrease in ERK1/2 phosphorylation and cyclin D1 levels and an increase in p27^Kip1^. Specific inhibition of BRAF by RNAi in cells with BRAF^V600E ^mutation had no effect on apoptosis. In the case of sorafenib treatment, cells harbouring BRAF^V600E ^mutation showed increase levels of apoptosis due to a balance of the anti-apoptotic proteins Mcl-1 and Bcl-2.

**Conclusion:**

Our results in thyroid cancer cells, namely those harbouring BRAF^V600E^mutation showed that BRAF signalling pathway provides important proliferation signals. We have shown that in thyroid cancer cells sorafenib induces apoptosis by affecting Mcl-1 and Bcl-2 in BRAF^V600E ^mutated cells which was independent of BRAF. These results suggest that sorafenib may prove useful in the treatment of thyroid carcinomas, particularly those refractory to conventional treatment and harbouring BRAF mutations.

## Background

A major event in the neoplastic transformation of thyroid follicular cells is the constitutive activation of a single signalling pathway, the RET/PTC - RAS - BRAF - MEK - ERK pathway. We, and others, have reported a high prevalence of BRAF point mutations (BRAF^V600E^) in papillary thyroid carcinomas (30% to 69%) and in anaplastic thyroid carcinomas (10% to 35%) [1,2]. In papillary thyroid carcinomas, BRAF mutations, RET/PTC rearrangements, and RAS mutations are mostly mutually exclusive [3].

In melanomas, which also harbour BRAF^V600E ^mutations, it has been demonstrated that BRAF^V600E ^activates the MAPK pathway and controls proliferation of melanoma cells through the regulation of cyclin D1 and of the cyclin-dependent kinase inhibitor p27^Kip1 ^[4-7]. Moreover, the suppression of BRAF^V600E ^in melanoma cells was demonstrated to inhibit proliferation, transformation, invasion and promote apoptosis [8-13].

In colon cancer suppression of BRAF in cell lines with BRAF^V600E ^showed significant decreased proliferation through cyclin D1 and p27^Kip1 ^and induces apoptosis by a significant decrease in the levels of anti-apoptotic protein Bcl-2 [14].

In thyroid carcinoma-derived cell lines, it was observed that inhibition of BRAF signalling by BRAF kinase inhibitors or BRAF RNAi inhibits growth, transformation and tumourigenicity of cell lines harbouring BRAF^V600E ^mutation, without any effect on apoptosis [15-19]. However, the molecular targets underlying the cellular effects induced by inhibition of the BRAF pathway in thyroid cells remain to be determined.

Using thyroid carcinoma cell lines, with different genetic profiles, we characterized the proliferation/survival associated molecules using RNAi targeting BRAF and the kinase inhibitor sorafenib, previously reported to inhibit BRAF [20].

## Methods

### Cell lines culture conditions

Cell lines - 8505C, and C643 derived from anaplastic thyroid carcinomas and TPC1 derived from papillary thyroid carcinoma - were grown in RPMI 1640 medium (with L-glutamine and HEPES) supplemented with 10% fetal bovine serum (FBS) and 1% penicillin-streptomycin (GIBCO, Invitrogen). All cells were grown in a humidified incubator with 5% CO_2 _at 37°C.

### BRAF RNA silencing

Small interference RNAs (siRNAs) sequences targeting human BRAF were designed according to Hingorani *et al *[8]: two of the oligos were specific for the V600E mutation (BRAF-mutA and BRAF-mutB) and two oligos recognize both the wild-type and mutated BRAF (BRAF-C1 and BRAF-C2). The control (non-silencing) siRNA used was that designed by Qiagen with the following target sequence: 5'-AATTCTCCGAACGTGTCACGT-3'. All siRNAs were purchased from Qiagen. Cells were transfected 24 hours after platting in 6-well plates in RPMI supplemented with 10% foetal bovine serum (FBS). Transfection was done using 3 μl of Lipofectamine 2000 (Invitrogen) and 50 nM of siRNA. Control cells were transfected with the siRNAs buffer alone. For the study of the uptake, cells were cultured in 6-well plates, trypsinized and fixed with 4% paraformaldehyde. Cytospin preparations were observed by fluorescence microscopy 24 hours after transfection with FITC-labelled siRNA (Qiagen). For confirmation of downregulation of BRAF protein, cells were seeded and transfected as indicated above and processed at 24, 48 and 72 hours.

### Drug treatment

Sorafenib stock solution was made at a concentration of 10 mM in DMSO and aliquots were kept at -20°C. Dose/response curves and IC50 doses were obtained by counting cell with trypan blue; briefly, cells were plated in 24 wells dishes and treated with increasing concentrations of sorafenib (0,4 uM, 2 uM, 4 uM and 10 uM) or vehicle (DMSO) in serum free conditions for different time points (24 h and 48 h). After treatment, cells in suspension and adherent cells were counted with trypan blue. After establishment of the dose range and optimal (IC_50_) sorafenib concentration (4 μM, 48 h of treatment), cells were plated in 6 well dishes at the appropriated cell density for proliferation (BrdU), apoptosis (TUNEL) and protein (Western blot) analysis.

### Apoptosis assay

Cytospin preparations of both floating and attached cells were collected and fixed with 4% paraformaldehyde (15 min) at room temperature. Cells were washed in PBS and permeabilized with 0.1% Triton X-100 in 0.1% sodium citrate (2 min) on ice. The TdT mediated dUTP Nick End Labeling (TUNEL) analysis was performed using the "In situ cell death detection kit, fluorescein" (Roche^®^), following the manufacturer's instructions.

### Assessment of DNA synthesis by BrdU incorporation

Cells were labelled by incubation in 10 μM bromodeoxyuridine (BrdU) for 1 h, fixed with 4% paraformaldehyde and nuclear incorporation was detected by immunofluorescence assay. The proportion of positive nuclei (BrdU index) was determined from a count of >500 cells.

### Western blot analysis

Cells were lysed for 5 min at 4°C using RIPA buffer (1% NP-40 in 150 mM NaCl, 50 mM Tris (pH 7.5), 2 mM EDTA), containing phosphatase and protease inhibitors. Proteins were quantified using a modified Bradford assay (Biorad). Protein samples (25 mg) were separated in 8%, 10% or 12% SDS/PAGE gels depending on the molecule to be analyzed and electroblotted to Hybond ECL membrane (Amersham Biosciences). The primary antibodies used were purchased from the following sources: anti-phospho p44/42 MAPK (Thr202/Tyr204), anti-p44/42 MAPK total were from Cell Signalling. Anti-BRAF, anti-Mcl-1, anti-p27, anti-CD1, anti-actin, anti-RAF-1 were from Santa Cruz Biotechnology. Anti-Bcl-2 was from Dako. Secondary antibodies (goat anti-rabbit and goat anti-mouse) were conjugated with peroxidase (Santa Cruz Biotechnology) and visualized by the ECL detection system (Amersham). Quantization of the specific signal was performed using Quantity One software from Bio-Rad.

### Statistical analysis

Two-tailed paired Student's *t*-test was used to perform statistical analysis. In all analysis *P *< 0.05 was required for statistical significance. Statistical analysis was done using StatView software program (PC version).

## Results

We tested the effect of BRAF inhibition by RNAi and sorafenib in cell lines representing the various genetic profiles found in thyroid tumours *in vivo*: 8505C harbours a homozygous BRAF^V600E ^mutation, TPC1 harbours a RET/PTC1 rearrangement and wild-type for BRAF, and C643 harbours a RAS^G13R ^mutation and wild-type BRAF [21], recently demonstrated to be unique thyroid cancer cell lines origin [22].

### Effect of BRAF inhibition by RNAi in proliferation and apoptosis of thyroid cancer cells

**Figure 1:**
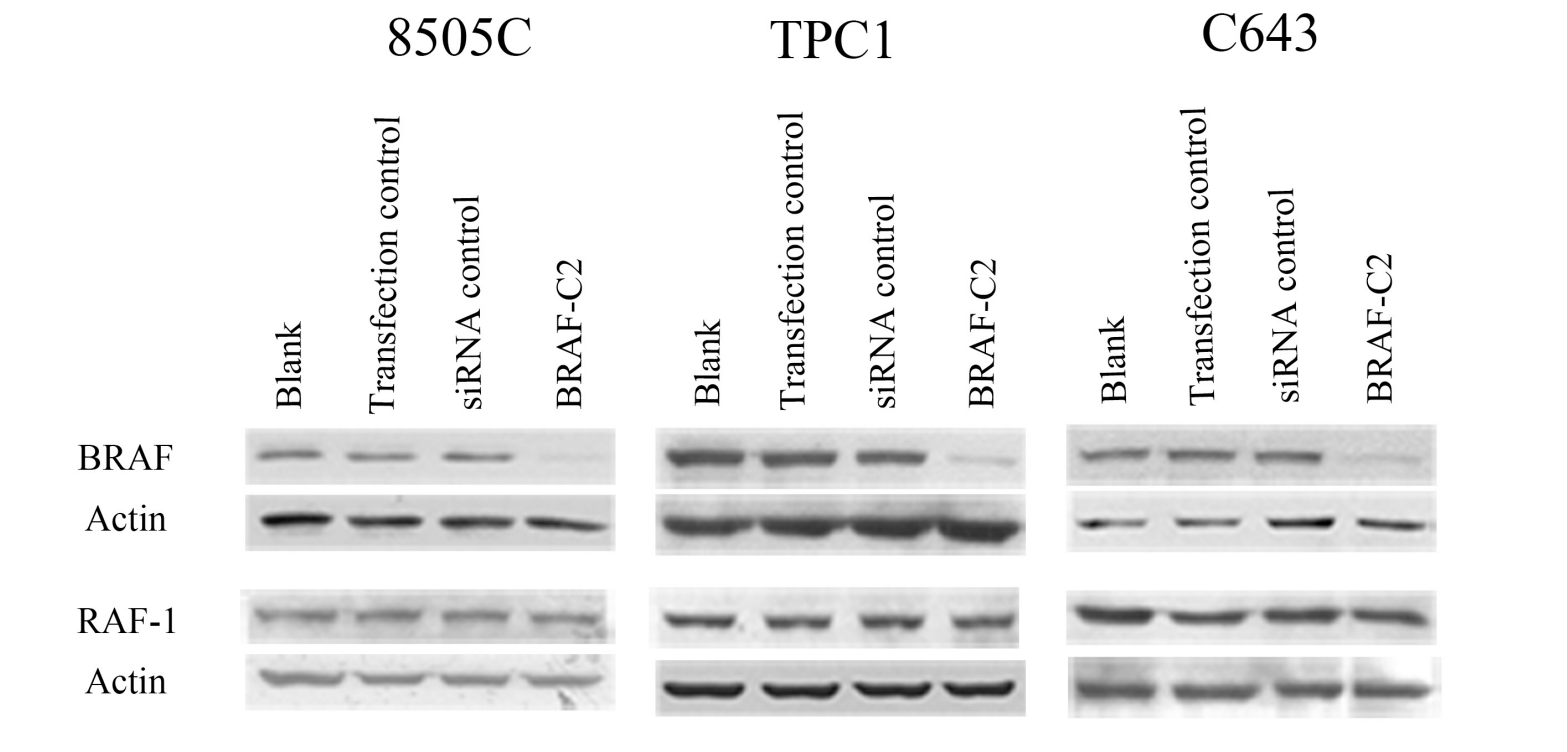
**Western-blot analysis of BRAF expression levels after RNAi treatment**. BRAF protein expression is inhibited in cells treated with siRNA BRAF-C2 in comparison to the blank, transfection control and siRNA control. Inhibition of BRAF was efficient in all cell lines. No alteration was seen in the levels of RAF-1 protein. The figure presented is representative of at least three independent experiments.

We used RNAi technology to specifically inhibit the BRAF gene. A panel of four BRAF siRNA oligonucleotides (mutA, mutB, C1 and C2) [8] were tested in all cell lines. After optimization (data not shown) the siRNA oligonucleotide BRAF-C2 which target both wild-type and mutated BRAF was selected, being the most potent at reducing BRAF levels after 48 hours of exposure. Transfection with BRAF-C2 siRNA led to inhibition of BRAF levels in all cell lines (Figure 1). No effect on the RAF-1 protein levels was observed using the BRAF-C2 siRNA (Figure 1).

**Figure 2:**
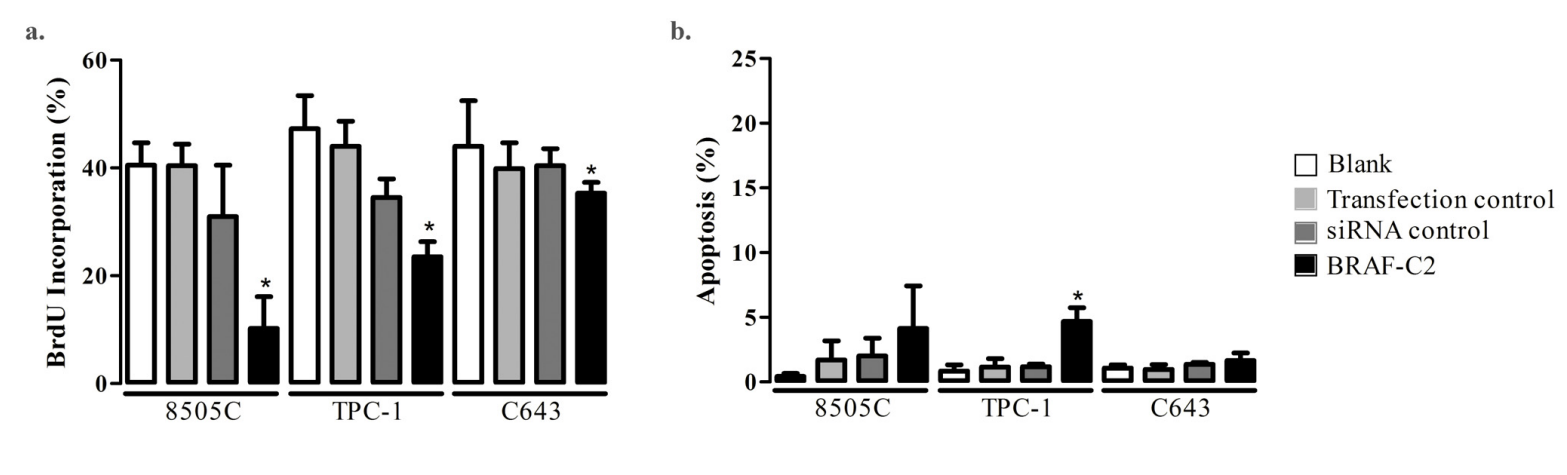
**BrdU and TUNEL analysis after RNAi treatment**. **(a) **BRAF knockdown using BRAF-C2 siRNA induces a significant (*p < 0.05) decrease in BrdU incorporation in comparison to the siRNA control in the three cell lines. **(b) **Inhibition of BRAF had no major effect on apoptosis as measured by TUNEL assay in 8505C and C643. In TPC1 cells there was a significant (*p < 0.05) increase in apoptosis when compared with the controls. All values presented are the mean of at least three independent experiments. In both, BrdU and TUNEL analysis, siRNA control did not produce any significant effect in comparison to the blank and transfection controls.

BRAF silencing induces a significant inhibition in the proliferation of all the cell lines (*p *< 0.05) in comparison to the siRNA control: 8505C (64.0%), TPC1 (37.9%), and C643 cell line (18.9%). In the case of the C643 cell line the decrease in proliferation was less pronounced but also reached the statistical significance. The effect was more evident in the cell line harbouring BRAF mutation (8505C) (Figure 2a).

The knockdown of BRAF induced a slight increase in apoptosis in 8505C cells (Figure 2b) as measured by TUNEL assay. In the TPC1 cell line, inhibition of BRAF led to a significant increase in apoptosis (Figure 2b). No significant effect on apoptosis was observed in the C643 cell line (Figure 2b).

In all cell lines analyzed the controls (blank, transfection control and siRNA control) were not significantly different between them for proliferation and apoptosis analysis (Figure 2).

### Effect of sorafenib in proliferation and apoptosis of thyroid cancer cells

Sorafenib a kinase inhibitor, developed as a RAF-1 kinase inhibitor [23,24] was shown to inhibit both the wild-type and the V600E mutant BRAF *in vitro *[20]. Sorafenib has been approved by the Food and Drug Administration (FDA) for therapy of renal cell carcinoma and is under clinical trials for melanoma and thyroid cancer [25]. A fixed dose of 4 μM of sorafenib for 48 h was selected since it represents the approximate IC_50_, for all cell lines (see Material and Methods).

**Figure 3:**
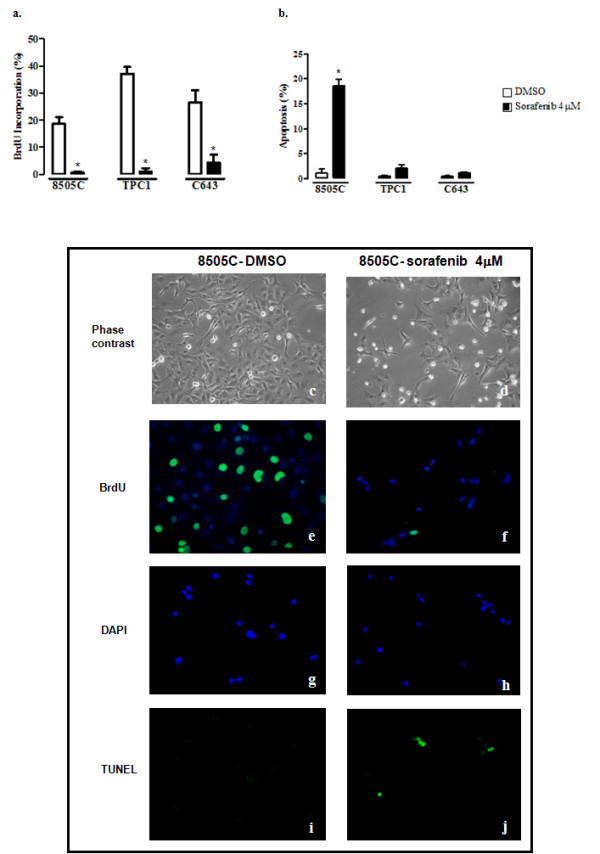
**BrdU and TUNEL analysis after sorafenib treatment**. Sorafenib inhibited the proliferation of thyroid carcinoma derived cell lines and induced apoptosis in the cell line harbouring the BRAF^V600E ^mutation (8505C). **(a) **Proliferation analysis by BrdU assay after treatment with sorafenib at a dose of 4 uM for 48 hours: all treated cell lines showed a significant (*p < 0.05) decrease in the percentage of cells incorporating BrdU in comparison to the vehicle (DMSO); **(e, f) **Representative photos of 8505C cell line showing decreased incorporation of BrdU in treated cells when compared with the DMSO control. **(b) **Apoptosis analysis by TUNEL assay after treatment with sorafenib at a dose of 4 uM for 48 hours. The cell line harbouring BRAF^V600E ^mutation (8505C) showed a significant increase (*p < 0.05) in the levels of apoptotic cells after sorafenib treatment in comparison to cell lines harbouring wild-type BRAF (TPC1, C643). **(g, h, i, j) **Representative photos of 8505C cell line showing increased TUNEL labelling in treated cells when compared with the DMSO control. DAPI: counter stain to detect nuclei in FITC-stained cells in TUNEL and BrdU assays **(c, d- phase contrast 20×; e, f, g, h, i, j, 40×)**. All values presented are the mean of at least three independent experiments.

Treatment with sorafenib induced a significant inhibition of proliferation (measured by BrdU incorporation) in all cell lines analyzed (*p *< 0.05) (Figure 3a and 3c-f). In comparison with the vehicle control, the percentage of cells incorporating BrdU diminished 96.9% in 8505C, 97.1% in TPC1 and 83.7% in C643 cell lines (Figure 3a).

To determine if the effect of sorafenib on cell growth was only due to inhibition of proliferation or in alternative was mediated by increased apoptosis, we performed a TUNEL assay. We observed an increase in the number of apoptotic cells in all the cell lines tested. In 8505C cell line there was a significant increase in apoptosis of 17 fold in comparison to the control (*p *< 0.05) (Figure 3b and 3c, d, g-j) whereas the level of apoptosis seen in TPC1 and C643 cell lines did not reach the level of statistical significance (Figure 3b). Sorafenib induces inhibition of proliferation in all cell lines and induces a significant increase in apoptosis in the cell line harbouring BRAF^V600E ^mutation (Figure 3). The results obtained in apoptosis with BRAF silencing suggest that the apoptotic effect mediated by sorafenib does not depend solely on the inhibition of BRAF.

### Effect of BRAF inhibition in ERK phosphorylation of thyroid cancer cells

**Figure 4:**
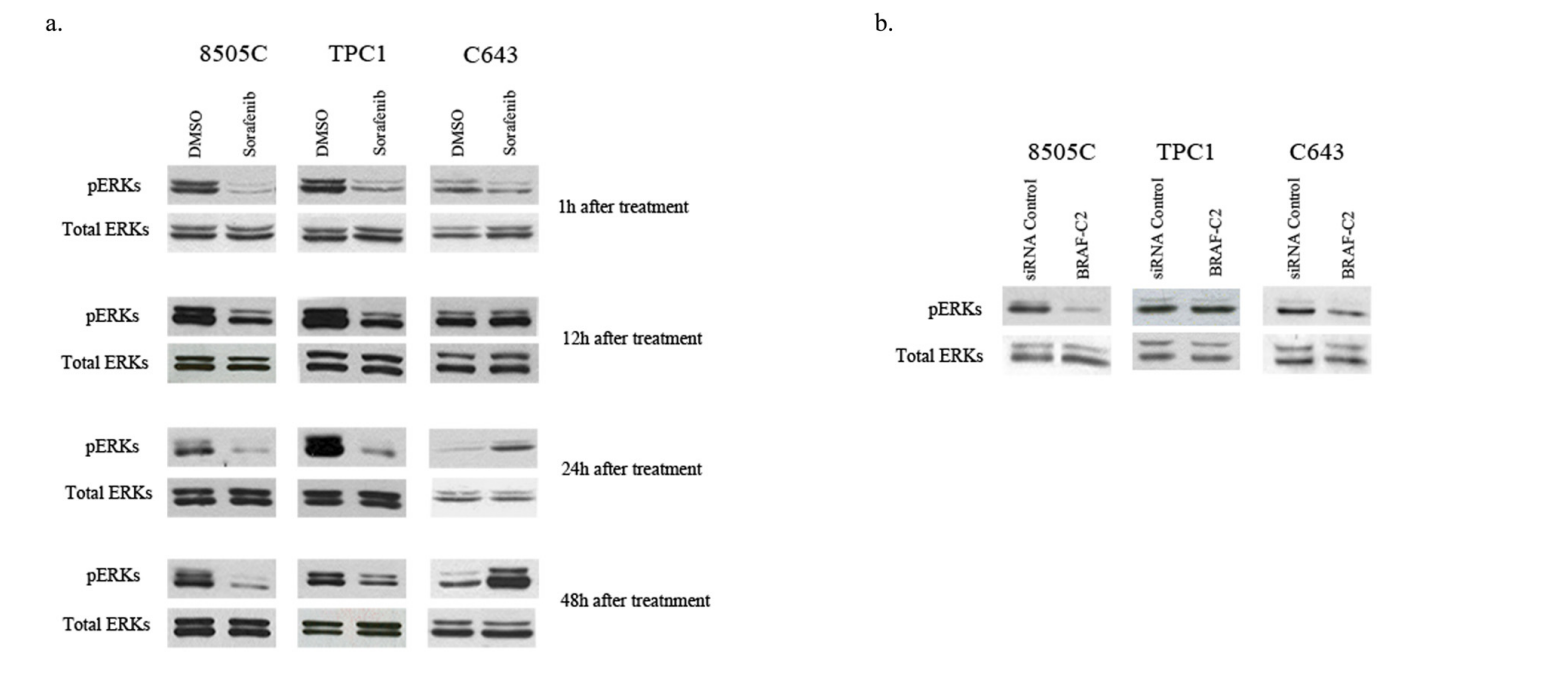
**Western-blot analysis of ERK phosphorylation levels after sorafenib and RNAi treatment**. **(a) **Sorafenib treatment leads to decreased levels of ERK phosphorylation. Cells were treated in serum free conditions with 4 uM of sorafenib or DMSO and proteins were extracted after 1 h, 12 h, 24 h and 48 h. The levels of phosphorylated ERKs after treatment were quantified relative to total ERKs and compared with the DMSO control. As shown in the graph, the levels of ERK phosphorylation decreased in all the cell lines at different time points, the levels were variable and transient in the time points analyzed in the different cell lines. **(b) **BRAF knockdown by RNAi induced decreased levels of ERK phosphorylation 48 h after transfection as detected by Western-blot analysis. Quantification of the levels of phosphorylated ERKs relative to total ERKs and compared with RNAi control (see graph), showed that BRAF-C2 siRNA inhibited ERK phosphorylation in 8505C and C643, but not in TPC1 cells. All values presented are the mean of at least three independent experiments.

BRAF specific inhibition by RNAi reduced ERK phosphorylation levels in 8505C and C643 cell lines, in comparison to the control siRNA (Figure 4b). In the TPC1 cell line (with a RET/PTC1 rearrangement) no differences were seen in the levels of phosphorylated ERKs (Figure 4b). Protein analysis revealed that sorafenib reduced ERK phosphorylation levels in all the cell lines after 1 h of treatment comparing with DMSO control (Figure 4a). ERK phosphorylation was variable and transient at the other time points analyzed (12 h, 24 h, 48 h) and in the different cell lines, as previously described by Ouyang *et al *[19] (Figure 4a).

### Analysis of target molecules implicated in the cellular effects induced by inhibition of BRAF pathway

We studied the expression of proteins involved in cellular proliferation (cyclin D1 and p27^Kip1^) and apoptosis pathways (pNFkB, pBad, Mcl-1, Bcl-2, Bax and XIAP) that are likely to be implicated in the cellular effects induced by inhibition of BRAF pathway either by RNAi or by sorafenib treatment.

#### Cell cycle related proteins analysis

**Figure 5:**
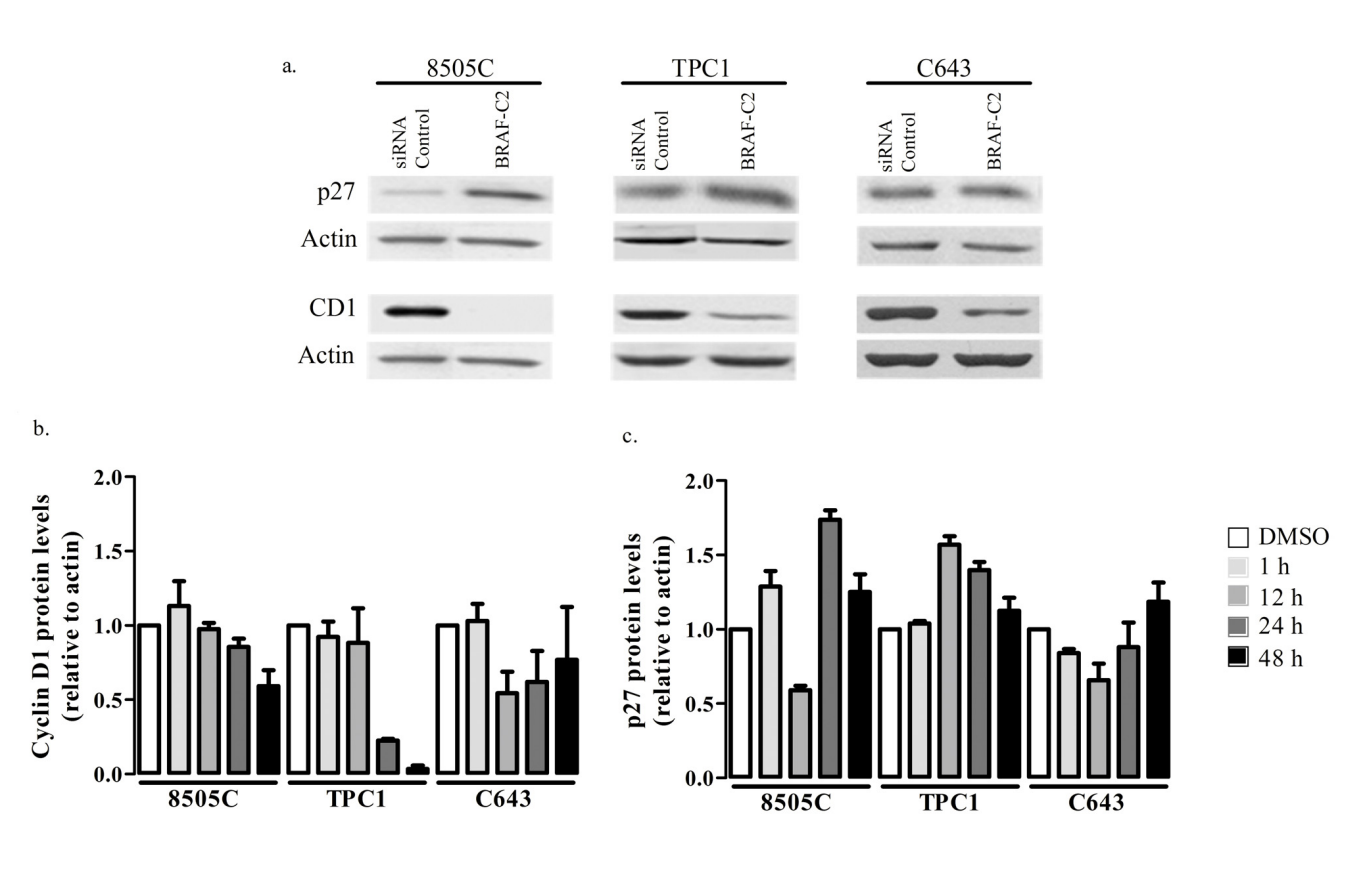
**Western-blot analyses of p27 and cyclin D1 expression levels after sorafenib and RNAi treatment**. **(a) **Inhibition of BRAF by BRAF-C2 siRNA decreased the levels of cyclin D1 in all the cell lines and increased the levels of p27^Kip1 ^in 8505C and TPC1 cells, but not in C643 cells as demonstrated in the Western-blot analysis and by quantification of the levels of cyclin D1 and p27 relative to actin (data not shown). **(b, c) **Sorafenib treatment induced alterations in cyclin D1 and p27^Kip1 ^at different time points depending on the cell line. The graphs represent the quantification of the levels of cyclin D1 and p27^Kip1 ^proteins relative to actin and compared with the DMSO control. **(b) **Treatment with 4 μM of sorafenib led to decreased expression of cyclin D1 at different time points and at different levels in all the cell lines. **(c) **Concerning the levels of p27^Kip1 ^after treatment with sorafenib: in 8505C cells there was an increase in all the time points except after 12 h; in TPC1 there is an increase after 12 h, 24 h and 48 h; in C643 there is an increase after 48 h. All values and figures presented are the mean of at least three independent experiments.

BRAF silencing by RNAi leads to inhibition of cyclin D1 expression in all the cell lines analyzed mostly in BRAF mutated cell line (8505C - Figure 5a). We also observed a pronounced increase in the levels of p27^Kip1 ^in 8505C and TPC1 and no apparent alterations in C643 (Figure 5a). These results suggest that the inhibition of proliferation observed after BRAF silencing is regulated through cyclin D1 and p27^Kip1 ^levels.

After treatment with sorafenib we observed inhibition of cyclin D1 expression in the three cell lines, exhibiting different inhibition kinetics at different time points analyzed (Figure 5b). With regard to p27^Kip1^we observed an increase of p27^Kip1 ^in 8505C and TPC1, whereas in C643 cells an increase was observed after 1 h and 48 h respectively, and an apparent decrease was observed in the other time points (Figure 5c).

#### Apoptosis related proteins analysis

**Figure 6:**
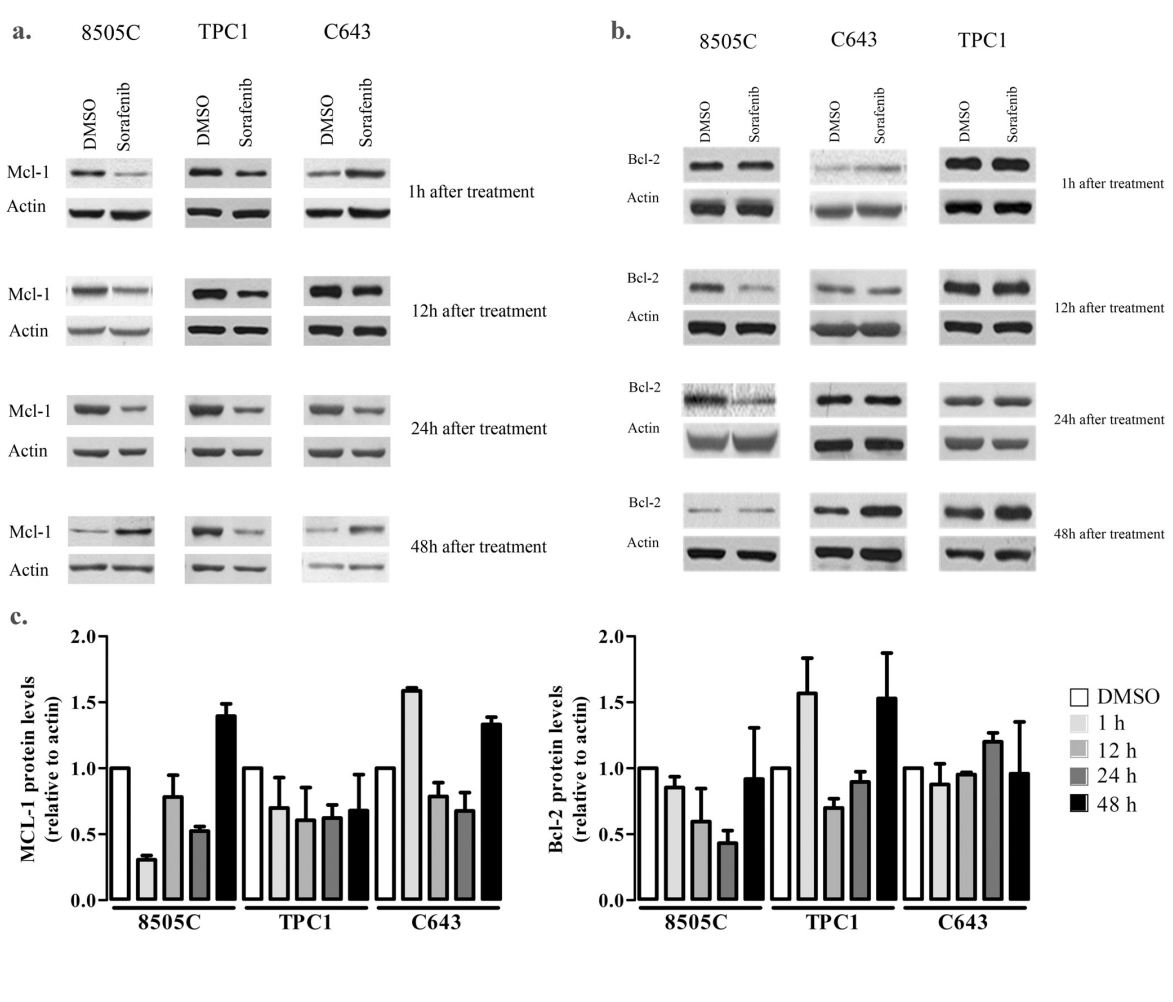
**Western-blot analyses of Mcl-1 and Bcl-2 expression levels after sorafenib treatment**. **(a) **Sorafenib treatment led to a marked decrease in the levels of Mcl-1 in the cell line with mutated BRAF^V600E ^(8505C) at all the time points analyzed except after 48 h in 8505C. Mcl-1 levels in TPC1 and C643 cells varied at the different time points after treatment, as shown in the graph (c): in TPC1 cells there was a decrease at all time points; in C643 cells there was a variable response with a increase seen at 1 h and 48 h. **(b) **Sorafenib induced alterations in the levels of Bcl-2 in the three cell lines. Levels of Bcl-2 protein were quantified, as shown in the graph (c): in 8505C cells the levels of Bcl-2 decreased at all time points; in TPC1 cells there was a marked increase after 1 h and 48 h; in C643 cells the levels of Bcl-2 remain unchanged at all time points except at 24 h where there was a increase. All values presented are the mean of at least three independent experiments.

Inhibition of BRAF by RNAi had no effect on the expression of Mcl-1 and Bcl-2 in the three cell lines (data not shown); this finding fits with the absence of significant differences detected in the apoptotic levels after transient inhibition of BRAF in the cell lines 8505C and C643. Although a significant increase in the level of apoptosis was seen in TPC1 cells (Figure 3b), none of the studied apoptotis related proteins showed alterations. Interestingly, the different cell lines treated with sorafenib showed different levels of Mcl-1 and Bcl-2 (two anti-apoptotic proteins). In 8505C cells, the decrease in Mcl-1 levels was transient, increasing after 48 h of treatment (Figure 6a). In C643 cells, we observed a decrease in Mcl-1 levels after 12 h and 24 h and an increase after 1 h and 48 h of treatment (Figure 6a). The decrease was more pronounced in the cell line with BRAF^V600E^. We also analyzed the expression of the anti-apoptotic protein Bcl-2 and observed that there was a pronounced decreased of Bcl-2 levels in 8505C cells after sorafenib treatment. In the remaining cell lines there was either no change, or only slight variations in the levels of Bcl-2 (Figure 6b). The balance in the levels of the anti-apoptotic proteins Mcl-1 and Bcl-2 overtime might be, in part, responsible for the different effects of sorafenib on apoptosis in 8505C cells, in contrast to TPC1 and C643 cells. The results obtained on Mcl-1 and Bcl-2 levels roughly parallel those obtained in the study of the levels of apoptosis in the same cell lines (compare Figure 3 and 6). The cell line that show a higher induction of apoptosis (8505C) by sorafenib is the one in which the decrease in the levels of Mcl-1 and Bcl-2 after treatment were more pronounced (Figure 6). No alterations were observed in the expression levels of other molecules implicated in survival pathways (pNFkB, pBad, Bax and XIAP) by RNAi or sorafenib treatment (data not shown)

## Discussion

BRAF a serine/threonine kinase, known to activate the MAPK pathway has been found mutated in several tumours, namely melanoma, colorectal carcinoma and thyroid carcinoma [3,4,26]. In thyroid carcinomas the molecules and pathways associated to the effect of BRAF inhibition in cellular proliferation and survival are not fully understood. We aimed to characterize in thyroid cancer cell lines with different genetic background those molecules implicated in proliferation/survival using RNAi targeting BRAF and the kinase inhibitor sorafenib.

Our results show that both approaches induce inhibition of proliferation in all the cell lines, regardless of the genetic background, although RNAi leads to a more pronounced effect in proliferation in BRAF^V600E ^mutated cell line. Our results support the hypothesis that thyroid cancer cells with activated BRAF are more dependent on the BRAF-ERK pathway for proliferation than those with RAS or RET/PTC1 activation.

BRAF is implicated in proliferation control through MAPK pathway downstream targets [27]. BRAF inhibition by RNAi strongly reduces ERK activation in the cell line harbouring the BRAF^V600E ^mutation, is also effective in the cell line with RAS^G13R^, but has no effect in ERK activation in the cell line with RET/PTC rearrangement. The higher levels of ERK inhibition achieved in the BRAF mutated cell line, by RNAi, demonstrates that in these cells BRAF is the main activator of ERKs. Our results in C643 cell line are in accordance with the activation of ERK proteins by activated RAS through BRAF dependent mechanism [28]. The absence of effect in ERK phosphorylation after BRAF inhibition by RNAi in TPC1 cell line suggests that RET/PTC1 activates ERK by a mechanism independent of wild-type BRAF. Our results suggest that RET/PTC1-mediated cell proliferation requires BRAF kinase but not BRAF-MAP kinase pathway. Mitsutake *et al *[29] have shown that, in a background of RET/PTC3 activation, BRAF is required for MAPK activation in PCCL3 cells. Our results, in TPC1 cell line, suggest that in a background of RET/PTC1 other molecules may signal to the MAPK pathway. In TPC1 cells we have previously suggested that RAF-1 could be the preponderant RAF isoform [30], and this might explain that specific inhibition of BRAF in this cell line does not have an effect on ERK activation at variance with RAS and BRAF mutated cells.

We showed that sorafenib inhibits efficiently ERK activation in all the cell lines regardless of the underlying oncogenic alteration. However, the inhibition of ERK phosphorylation secondary to the treatment with sorafenib was variable and transient in the different cell lines, as previously demonstrated by Ouyang *et al *using other RAF kinase inhibitors [19]. This variation between cell lines can be associated to its effect against angiogenesis-related receptor tyrosine kinases such as VEGFR -2 and -3, along with other kinases such as PDGFR-β, Flt-3 and c-Kit [20,23,24,31,32]. The level of ERK inhibition achieved in TPC1 by sorafenib, and not by BRAF RNAi, indicates that sorafenib targets other molecule(s) besides BRAF, namely RET/PTC oncogenic protein itself, as previously advanced [23,31].

The results we obtained with BRAF siRNA in cells with mutated BRAF (8505C) show that BRAF^V600E^-ERK signalling is important in the regulation of proliferation. Several proteins have been shown to be targeted by the BRAF/MEK/ERK signalling pathway in distinct tumours models (melanoma, colorectal and thyroid carcinomas); those proteins include cyclin D1 and p27^Kip1^, implicated in cell cycle regulation [6,7,14,33]. In normal thyrocytes cyclin D3 is the predominant D-type cyclin, but in papillary carcinoma cells with BRAF mutation cyclin D3-CDK4 activation is lost [30]. In thyroid cancer cell lines with RET/PTC or BRAF oncogenic mutations cyclinD1-CDK4 complex is more abundant than cyclinD3-CDK4, suggesting the dominant character of cyclin D1 complexes formation in thyroid oncogenesis [30]. This is in accordance with our results showing that, in thyroid cancer cell lines a decrease in proliferation parallel with a decrease in cyclin D1 levels. Our results showed, furthermore, that in cells with RET/PTC1 rearrangement, inhibition of BRAF by RNAi besides decreasing cyclin D1 levels, increases the levels of p27^Kip1 ^and leads to the inhibition of proliferation, which was independent of ERK inhibition. In a background of RAS activation (C643 cell line), BRAF inhibition decreases the levels of phosphorylated ERKs and cyclin D1 and has no effect on the levels of p27^Kip1^. This results support that p27^Kip1 ^might be regulated directly by activated RAS, as previously advanced by Jones *et al *[34].

The variability observed in the expression levels of cyclin D1 and p27^Kip1 ^in all cell lines after treatment with sorafenib, may also result from the ability of sorafenib to functioning as a multikinase inhibitor [20,23,24,31,32].

We have shown that selective downregulation of BRAF does not induce apoptosis in thyroid cells with BRAF mutation at variance with sorafenib that induces marked apoptosis in BRAF^V600E ^mutated cells. In melanoma cell lines it was shown that sorafenib treatment can induce cell death, leading to Bad dephosphorylation, decrease in the levels of Bcl-2 and Bax activation [11,35]. In addition, sorafenib has also been shown to downregulate the prosurvival Bcl-2 family member Mcl-1 which may be a mechanism through which the compound mediates its pro-apoptotic effect [36]. Our results suggest that in thyroid cells with BRAF^V600E ^oncogenic activation the effect of sorafenib in apoptosis depends upon the balance in the levels of the anti-apoptotic proteins Mcl-1 and Bcl-2 and not in the levels of Bad and Bax (data not shown), as previously demonstrated in melanoma cells [11,35,36]. We have also shown that NFkβ and XIAP do not seem to be involved in that process. The most striking downregulation of the levels of Mcl-1 and Bcl-2 proteins was observed in the cell line harbouring mutated BRAF (8505C) in which sorafenib induced also the highest levels of apoptosis. These results fit with previous reports in melanoma cell lines [35,36]. These effects were not observed after BRAF specific inhibition by RNAi, suggesting a BRAF-independent mechanism for sorafenib in the regulation of Mcl-1 and Bcl-2 expression and induction of apoptosis, in cells with mutated BRAF. Inhibition of BRAF by siRNA induces apoptosis only in TPC1 cells harbouring RET/PTC1 indicating that wild-type BRAF seems to be important in survival of these cells.

The mechanism by which sorafenib downregulated the levels of Mcl-1 is likely to depend on enhanced proteosome-mediated Mcl-1 degradation [36]. This downregulation of Mcl-1 by sorafenib is not cell type dependent or selective for BRAF-mutated cell lines since this effect was observed in all cells lines analysed some of them without BRAF mutation [35,36]. The mechanism by which sorafenib downregulates the levels of Bcl-2 is not well understood. This mechanism seems to be cell dependent as it was only observed in melanoma cell lines by Yu C *et al *[36] and in thyroid carcinoma cells by us in the present study. Bcl-2 downregulation in melanoma cells after sorafenib treatment occurs in cell lines harbouring BRAF mutation but seems to be ERK-independent [36]. However, in our study we observed a more pronounced decreased of Bcl-2 levels in BRAF mutated thyroid cells which was dependent on ERK activation.

## Conclusion

In this study we described for the first time, to the best of our knowledge, the effect of both sorafenib and specific siRNA for BRAF in thyroid cancer cells and associated molecules. Our results show that BRAF plays a major role in the proliferation of thyroid carcinoma cells independently of the oncogenic activation, suggesting a role of wild-type BRAF also in RET/PTC and activated RAS signalling pathways. Our results also show that p27^Kip1 ^and cyclin D1 proteins are important in the regulation of proliferation *via *BRAF^V600E^-ERK signalling and BRAF does not seem to be a major protein for the survival of thyroid cancer cells.

Treatment of thyroid carcinomas is usually achieved through the use of radioactive iodine (^131^I). Although the majority of thyroid carcinomas respond well to radioiodine therapy, there are thyroid tumours resistant to this therapy, that are inoperable and have lost radioactive avidity [25]. Taking into account the high prevalence of BRAF mutations in thyroid tumours it is tempting to consider the use of BRAF inhibitors as a therapeutic approach in these cancers. A recent phase II clinical trial of sorafenib showed a significant anti-tumour activity in advanced thyroid cancer without molecular characterization [37]. Our results indicate that sorafenib might be particularly potent in thyroid tumours harbouring BRAF mutations since, in addition to inhibition of proliferation it is also able of inducing apoptosis in these settings.

## Competing interests

The authors declare that they have no competing interests.

## Authors' contributions

AP conceived the study and its design, performed the analysis and interpretation of data and drafted the manuscript. JG carried out the RNA interference studies, draw the graphs and figures and performed the statistical analysis. APR carried out the sorafenib studies. JF participated in the RNA interference studies. AMM participated in the establishment of sorafenib doses to be used in the study. ASR made important contributions in the analysis and interpretation of data. HMV and HS participated in the optimization of the RNA interference conditions to be used in the cell lines. RS participated in the interpretation of data and revised critically the manuscript. PS conceived the study and its design, participated in the analysis and interpretation of data and helped in the draft of the manuscript. MSS was responsible for the study coordination or for revising critically the manuscript putting important intellectual contents. All authors read and approved the final manuscript.

## Pre-publication history

The pre-publication history for this paper can be accessed here:

http://www.biomedcentral.com/1471-2407/9/387/prepub
